# How Neurotech Start-Ups Envision Ethical Futures: Demarcation, Deferral, Delegation

**DOI:** 10.1007/s11948-022-00421-1

**Published:** 2023-02-02

**Authors:** Sophia Knopf, Nina Frahm, Sebastian M. Pfotenhauer

**Affiliations:** 1 School of Social Sciences and Technology, Department of Science, Technology and Society, Technical University Munich, Munich, Germany; 2 School of Management, Technical University Munich, Munich, Germany; 3 School of Communication and Culture, Department of Digital Design and Information Studies, Aarhus University, Aarhus, Denmark

**Keywords:** Neuroethics, Responsible innovation, Direct-to-consumer neurotechnology, Vanguard visions, Knowledge-control regimes, STS

## Abstract

Like many ethics debates surrounding emerging technologies, neuroethics is increasingly concerned with the private sector. Here, entrepreneurial visions and claims of how neurotechnology innovation will revolutionize society—from brain-computer-interfaces to neural enhancement and cognitive phenotyping—are confronted with public and policy concerns about the risks and ethical challenges related to such innovations. But while neuroethics frameworks have a longer track record in public sector research such as the U.S. BRAIN Initiative, much less is known about how businesses—and especially start-ups—address ethics in tech development. In this paper, we investigate how actors in the field frame and enact ethics as part of their innovative R&D processes and business models. Drawing on an empirical case study on direct-to-consumer (DTC) neurotechnology start-ups, we find that actors engage in careful boundary-work to anticipate and address public critique of their technologies, which allows them to delineate a manageable scope of their ethics integration. In particular, boundaries are drawn around four areas: the technology’s actual capability, purpose, safety and evidence-base. By drawing such lines of demarcation, we suggest that start-ups make their visions of ethical neurotechnology in society more acceptable, plausible and desirable, favoring their innovations while at the same time assigning discrete responsibilities for ethics. These visions establish a link from the present into the future, mobilizing the latter as promissory place where a technology’s benefits will materialize and to which certain ethical issues can be deferred. In turn, the present is constructed as a moment in which ethical engagement could be delegated to permissive regulatory standards and scientific authority. Our empirical tracing of the construction of ‘ethical realities’ in and by start-ups offers new inroads for ethics research and governance in tech industries beyond neurotechnology.

## Introduction

2020 was the year that neuroethics debates undoubtedly made a splash in the private sector. In August, *IBM Research* director Dario Gil urged readers of the *Scientific American* to "act now to avoid any future risks as neurotech matures—for the benefit of humanity" (Gil, 2020). Gil’s call to action followed on the heels of Elon Musk describing the high-bandwidth brain-machine-interface (BMI) technology, launched by his company *Neuralink* just a year earlier, as "for sure [..] an ethics first situation" (Swisher, 2020). *Facebook*—having just announced the acquisition of *Ctrl-Labs*, a start-up that produces non-invasive brain-computer-interfaces (BCIs) in the form of wristbands—publicly stated that “neuroethical design” was one of the “key pillars" of its virtual reality program (Facebook, 2020). Pushing the frontier of minds and machines, Big Tech and its visionaries have not only become eager to make their bold promises become reality but increasingly also underline the importance of doing so in an ethical way.

The statements by IBM, Neuralink or Facebook reflect a growing tension between the potential social benefits of neurotechnology innovation driven by companies on the one hand, and its ethical, legal, and social ramifications on the other—especially in the context of rapid large-scale commercialization (Garden et al., 2019; Garden & Winickoff, 2018; Pfotenhauer et al., 2021). Such tensions are closely observed by policy-makers across the world and have provoked a number of proposals on how to govern emerging neurotechnology in ethically sound ways (Greely et al., 2018; Salles & Farisco, 2020; Yuste et al., 2017). A recent policy recommendation released by the Organisation for Economic Cooperation and Development (OECD, 2019), for example, encourages building a "culture of stewardship and trust in neurotechnology across the private and public sector" (p. 8). However, as argued by some analysts, existing approaches to the integration of ethics have been found rather ineffective (if not inapplicable) in business settings. While frameworks for neuroethics and responsible innovation have been successfully mobilized within the governance of public neurotechnology R&D (Garden & Winickoff, 2018; Nuffield Council on Bioethics, 2013), instruments for corporate responsibility in the private sector rarely focus on technological innovation as a key activity of businesses today (Pfotenhauer & Frahm, 2019; Birch et al., 2017). This apparent gap between private and public sector options for neuroethics and governance represents a challenge for many actors, and needs to be addressed so as to ensure emerging neurotechnology R&D is translated responsibly into societies.

Beyond Big Tech’s efforts to scale up BCI technology, neuroethics debates have been particularly prominent regarding *direct-to-consumer (DTC) neurotechnology* (Garden et al., 2019; IEEE, 2019; Wexler & Reiner, 2019), commonly understood as neurotechnology products that are marketed directly to healthy individuals and for non-medical purposes (Ienca & Vayena, 2019; Ienca et al., 2018). While driven by a flourishing start-up community, discussions about ethics in DTC neurotechnology at present remain primarily shaped by expert communities from outside of the private sector (Sample et al., 2020) and currently lack the important perspective of start-ups when it comes to deliberating the integration of ethics into engineering, design, and business practices. Both scholars and practitioners have noted a certain disconnect of available neuroethics expertise and start-up needs: As Coates McCall et al. (2019) have argued, "empirically driven knowledge about the ethical realities of commercializing [DTC neurotechnology] devices in the open marketplace is limited” (p. 728). What is more, despite their increasing commercialization, innovative DTC neurotechnologies developed by start-ups often fly under the radar of public and regulatory scrutiny mostly aimed at medical devices and public research initiatives.

Taking the private sector as an arena for neuroethics seriously, this paper explores how start-ups in the domain of DTC neurotechnology reason about and construct the ethical realities of their innovations, and what kind of politics become visible once the keyword of "ethics" is "unpacked" in this context (Moss, 2020). Rather than proposing how DTC neurotechnology start-ups *ought* to address ethics—e.g. based on some *ex-ante* understanding of ethics or through instrumental toolkits—we investigate empirically how technology start-ups enact ethics "bottom-up” (Racine, 2010, p. 71). We draw on interviews, ethnographic observation and extended document analysis to show how actors actively construct and police what counts as ethics through careful acts of *boundary-work* (Gieryn, 1983) in four domains: the technology’s actual capability, purpose, safety and evidence-base. Our analysis is grounded in the co-productionist strand of Science and Technology Studies (STS; Jasanoff, 2004) that has been fruitfully applied to empirically grounded neuroethics in the past (Sample et al., 2020). We argue that the observed strategies of boundary-work contribute to an understanding of ethics that is tied to a specific distribution of ethical responsibilities and that is co-produced with particular *vanguard visions* about plausible and desirable neuro-futures (Hilgartner, 2015). Through such visions, neurotech entrepreneurs chart technology trajectories and corollary ethical obligations as a relationship between the present and the future. Here, the future figures as a promissory site where neurotech's benefits will have materialized and to which the resolution of complex ethical issues can be deferred. Conversely, ethical engagement in the present is seen as subject to permissive regulatory standards and underutilized scientific authority. This framing of ethics, as we suggest, allows the actors of our research to navigate the regulatory and ethical uncertainty surrounding neurotechnological innovation and establish legitimacy, credibility and autonomy for actors and organizations vis-à-vis potential public concerns.

Our approach and analysis offers new inroads for future research on the governance and ethics of innovation as well as potential for intervention into the space where it is arguably most needed—the private sector and specifically start-ups—with possible links to debates around *neuroethics* (Farah, 2010; Racine, 2010), *Responsible Research and Innovation* (Stilgoe et al., 2013; Pfotenhauer et al., 2021) and *Corporate Social Responsibility* (Crane et al., 2008). Following our findings, we suggest that approaches to the governance of innovation that meaningfully incorporate ethical responsibility in consumer neurotechnology and beyond need to be attentive to the politics of key actors, such as start-ups, in constructing the ethical realities and futures of their socio-technical interventions.

## Problematizing the Ethics of Direct-to-Consumer Neurotechnology

### A Short Overview of Direct-to-Consumer Neurotechnology

Exploring the need for ethical deliberation in emerging neurotechnologies, neuroethicist Giordano (2012) defines neurotechnology as “devices that are utilized to investigate, assess, access, and manipulate the structure and function of neural systems” (p. 4) which include interventions as diverse as pharmaceuticals, implants, recording technologies (e.g., EEG, fMRI), stimulating technologies (e.g., deep brain stimulation), and mental health apps. While many such technologies are aimed at medical diagnosis or treatment, neurotechnology increasingly makes its way into consumer products (Ienca et al., 2018). Ienca and Vayena (2019) noted that the boundaries circumscribing *direct-to-consumer (DTC) neurotechnology* are fluid and contested. Looking at the literature, the term most commonly denotes non-invasive wearable neurotechnology devices (Kellmeyer, 2018) that are purchasable directly by consumers (Kreitmair, 2019), and sold on the consumer market for non-medical purposes and to healthy individuals (although we will see the difficulties of such demarcations in our discussion below). Such devices are offered under the rubrics of "wellness, wellbeing and human flourishing" (Ienca & Vayena, 2019, p. 150), including enhancement of cognitive ability or attention, meditation, stress relief, weight loss, improving sleep, and controlling virtual reality settings for gaming or educational purposes (e.g., Coates McCall et al., 2019). Currently available products, for instance to improve sleep or meditation, often include a device (e.g., a headset) that records brain activity via electroencephalography (EEG) as well as an app that lets users review their measurements, learn to adjust their mental state, and access programs and reports. Other products use electrodes that stimulate selected areas of the brain via transcranial direct or alternating current stimulation (tDCS/tACS) in order to temporarily increase brain plasticity and enhance, for instance, movement or working memory.

The question whether a neurotechnology device is a medical product has considerable regulatory implications, including what marketing claims can be made about the product. From a technological perspective, the boundary between DTC and medical neurotechnology is inevitably blurry, as “DTC neurotechnologies exist on a spectrum” (Kreitmair, 2019, p. 155) ranging from technologies that are visibly non-medical, e.g. for gaming, to others which could technically be employed for medical purposes, such as certain wearables. Across this spectrum, both the intended purpose of and respective claims made about the product play an important role in determining whether a novel neurotechnology has to undergo the regulatory pathway as a medical product.^1^ Manufacturers must thus navigate a fine line between stating that a product can, for instance, ‘cure’ or ‘treat’ a medical condition or just ‘support a healthy lifestyle’. While medical devices undergo lengthy, diligent and expensive processes of regulatory approval (e.g., by the Food and Drug Administration (FDA) in the US or Notified Bodies across the EU) in which the manufacturer must evidence a product’s safety and efficacy with clinical data, consumer products are subject to much less cumbersome oversight. This is also the case for the FDA's definition of “low risk general wellness devices” (FDA, 2019) which some DTC neurotechnology products could be classified as.

In contrast to a clear set of medical and research ethics rules for medical devices (e.g., World Medical Association, 2013), regulations regarding what constitutes ethical R&D are largely absent for consumer products. As Wexler and Reiner (2019) observe, "there are good reasons to conclude that regulatory oversight of Direct-To-Consumer neurotechnologies is insufficient” (p. 234), stoking fears of unethical behavior and a tendency of actors to push the promissory boundaries of innovative neurotech. The absence of regulation and oversight in DTC markets represents a dilemma for many companies. On the one hand, it provides some leeway to choose which market they want to enter, what evidence they want to provide, and which rules they want to obey (Wexler, 2015). On the other hand, given the absence of clear criteria by which they could demonstrate their ethical integrity, it can lead to uncertainty amongst industry actors about what counts as ethical behavior in the quasi-unregulated space of DTC neurotechnology, and potentially threaten the future of emerging products and markets.

Regulatory and ethical ambiguity is particularly pressing for start-ups who at present are arguably the most important actors pushing innovative neurotechnology forward (Garden et al., 2019; Wexler & Reiner, 2019; Pfotenhauer et al., 2021). Entrepreneurial culture, and its embodiment of the Silicon Valley, not only became (in)famous for bold visions, but also for creative solutions and a pioneering spirit of "move fast and break things" (Taplin, 2017). As characterized by Metcalf et al. (2019), "Silicon Valley logics hold that trenchant social problems can be addressed through innovative technical solutions […] developed by those with the most aptitude and creative energy […], and that an unencumbered market will recognize, reward, and disseminate the best solutions […]” (p. 460). Reflecting Schumpeterian ideas of market competition, these logics “underwrite business as usual at the same time as they are implicated in many industry approaches to ‘doing ethics’" (ibid., p. 461).

On the downside of speed and scale, technology start-ups have been criticized for their affinity to risk, considering negative concomitants a productive part of the process (Maynard & Garbee, 2019) and for making fast growth their first priority (Pfotenhauer et al., 2021; Cook, 2020). As part of a recent scandal, *Lumos Labs* stated that their app Lumosity could improve cognitive performance or protect against cognitive decline. It was fined with $2 million following accusations of deceptive advertising using unsubstantiated health claims, which were later prohibited by the U.S. Federal Trade Commission (Federal Trade Commission, 2016). The case exemplifies the challenges that neurotechnology start-ups currently face—from making rightful claims and selling a novel product to arising scrutiny by regulators and the public—emerging from the interplay of competition regarding speed and funding from venture capital (VC), a tendency to regulatory iconoclasm, and the uncertainty regarding marketing strategies and claims.

### Governance Challenges of Direct-to-Consumer Neurotechnology

Among the different responses to governance challenges surrounding emerging neurotechnologies, *neuroethics* (Farah, 2010) is probably the most widespread discourse in which ethics deficits and governance options are currently discussed. Neuroethical concerns range from issues such as safety, privacy of brain data (Kellmeyer, 2018), informed consent (Racine & Aspler 2017), and an exacerbation of social inequality through cognitive enhancement (Kelly & Ford, 2015), all the way to questions of free will, autonomy and personal identity (Racine & Aspler 2017; Costa, 2010). The governance of DTC neurotechnology seems to be particularly prone to some of the noted challenges. Scholars have criticized that commercially available consumer products lack regulatory oversight and have the “least stringent requirements for accuracy and reliability” (Kreitmair & Cho, 2017, p. 88), and suggested that “companies in this market sector must be alert to all of the ethical and social impact of their R&D and marketing activities and be prepared to incorporate these issues into business development plans” (Eaton & Illes, 2007, p. 396). Further issues are attributed to its commercial nature, such as processes of data accumulation and confidentiality (Eaton & Illes, 2007; Ienca et al., 2018; Kellmeyer, 2018) as well as the lack of a scientific foundation of marketing claims (Wexler & Thibault, 2019), their potentially misleading nature regarding accuracy (Wexler & Reiner, 2019), efficacy or health benefits (Coates McCall et al., 2019). The specific issues that the neuroethics discourse addresses are closely intertwined with an understanding of the brain as the primary locus of human identity and agency, as well as the increasing pervasiveness of neuroscience and -technology in all areas of life (Rose & Abi-Rached, 2013)—often referred to as the ‘neuro-turn’. Connected philosophical, psychological and legal questions have rekindled ethical debates about neuro-essentialism, i.e., whether neuroscience and neurotech indeed raise unique ethical issues (e.g. in comparison to other domains of bioethics) that require unique ethical responses (Racine & Sample, 2019).

Since the rise of large-scale public research initiatives such as the EU Human Brain Project and the U.S. BRAIN Initiative, questions of ethics in neurotechnology also became a concern for policymakers, constituting a second noteworthy strand of the debate. A significant body of work centers on the concept of responsible innovation which has been institutionalized through the public policy framework *Responsible Research and Innovation* (RRI) and which has been suggested for the governance of public sector neurotechnology R&D in general as well as to medical applications (e.g., Nuffield Council on Bioethics, 2013; The Royal Society, 2011). As a result of this attachment to the public sector, particular concerns of businesses and start-ups, and questions specific to DTC neurotechnology, have received considerably less RRI attention. A recent noteworthy exception is the OECD report on *Responsible Innovation in Neurotechnology Enterprises* (Garden et al., 2019) that considers the growing role of start-ups in neurotechnology as well as the emerging consumer market. The report’s key findings include that consumer-oriented devices need to “comply with relevant standards in terms of safety, efficacy and interoperability with clinically-graded equipment “ (p. 27). In another recent paper, Pfotenhauer et al. (2021) suggest the need to co-develop shared governance frameworks for private sector neurotechnology innovation by including both the private and public sector in the process early on.

Lastly, drawing on a broader debate around the limitations of existing governance approaches for the integration of ethics into private sector innovation and especially start-ups, there has been renewed scholarly attention to frameworks of *Corporate Social Responsibility (CSR)* and their applicability to technology companies (e.g., Iatridis & Schroeder, 2016). In that context, frameworks for corporate governance that have historically integrated considerations of ethics into the private sector have been found limited regarding their potential to inform tech ethics in the "innovation era" (Pfotenhauer et al., 2019). Pfotenhauer and Frahm (2019) have argued that CSR focuses on specific aspects of corporate conduct and compensation rather than questions of innovation governance, and thus provides only little guidance on how to responsibly implement emerging technologies into society as a company. This is especially noteworthy regarding start-ups: While employing CSR tools in MNEs and corporations is daily business, start-ups often lack the organizational and financial resources. Metcalf et al., (2019) point out that start-ups grow before they mature organizationally, and hence ethics are often integrated post hoc rather than being embedded in the very fabric of the businesses.

## Unpacking 'Ethics' in Direct-to-Consumer Neurotechnology Start-Ups

In the following, we explore how neurotech companies cope with the thorny ethical terrains they are facing. We apply an empirically grounded, pragmatist and constructivist approach to ethics compatible with the methodological toolkit of Science and Technology Studies (STS). Our approach deviates from parts of the *neuroethics* literature, which has primarily taken a normative and instrumental stance that focuses on the lack of ethics integration in the translation of neuroscience into marketable products. So far, little attention has been paid to what the neurotechnology industry itself understands as an ethical issue and how they deal with it, with empirically grounded insights into how the industry navigates different positions regarding what is good and right in the field in light of their practices being “under fire” (Schiølin & Frahm, forthcoming) largely missing. Our work is part of a larger push towards more empirically informed pragmatist approaches in neuroethics (Racine, 2010; Sample et al., 2020) and processes of generating policy solutions that “involve representation of key stakeholders to maximize both trust and trustworthiness” (Lázaro-Muñoz et al., 2019, p. 98).

Following STS scholar Steven Hilgartner (2015), we understand DTC neurotechnology start-ups as sociotechnical *vanguards* who propose certain revolutionary *visions* of desirable futures shaped by their novel technologies. By showing that the actors’ framing and enactment of ethics is part of their strategy of establishing plausible and favorable *vanguard visions* of technology trajectories from the present into the future, we will argue that ethics is not an *a priori* given. Instead, it emerges as the result of a complex political positioning and negotiation by the start-ups we investigated that is tied to considerations of operationalizability and situated in the entrepreneurial start-up culture.

In particular, we make use of the concept of *boundary-work* to show how start-ups construct ethical vs. unethical neuro-innovation, including what is envisioned as ethical innovation and business practice in the future and what is perceived as ethical challenge of the present. Studying discourses of scientists on science, Gieryn (1983) argued that "boundary-work describes an ideological style found in scientists' attempt to create a public image for science by contrasting it favorably to non-scientific intellectual or technical activities" (p. 781), which serves as a discursive strategy to preserve the autonomy and integrity of actors. Jasanoff (1987) interrogated the critical function of boundary-work for "regulatory science" in situations where scientific expertise is mobilized for regulatory decisions, including the need for ethical intervention. Tracing the conflicts that may ensue between different groups and self-interested ways in which issues are declared "scientific", "political" or "ethical", she describes how “actors use boundary-defining language in order to distinguish between science and policy, and to allocate the right to interpret science in ways that further their own interests” (p. 195). Our approach follows such insights and extends them further to current demarcation mechanisms in the field of ethics in the technology sector and through start-ups in particular: What is described as ethical innovation by company leaders and staff, how do they differentiate between ethical and unethical business practices and products, and which vision of desirable neuro-innovation emerges from their reasoning around ethics?

### Empirical Material and Methods

Following a qualitative and interpretative approach, our analysis draws on 14 semi-structured interviews (conducted between July and November 2019) with actors in the DTC neurotechnology field. In particular, the group of interviewees consisted of 12 members of companies (both on the executive and staff level) and two participants who we addressed as experts of DTC neurotechnology. Due to their work, they were deeply immersed into the field without being affiliated with a specific company at that time. We defined the eligibility of organizations through two criteria: 1) They needed to work on products in line with the understanding of DTC neurotechnology presented above, and 2), as this research project looks at start-ups and emerging businesses, larger established firms were excluded. All names of the interview participants as well as the affiliated organizations that would allow any form of identification were anonymized. Further empirical sources, such as documents, news media articles, social media channels or webinars in the neurotechnology scene, as well as ethnographic observations, were included during research; those were not approached as systematically but, similar to an approach Wexler (2016) took in researching do-it-yourself neurotechnology, served as an "impressionistic sketch" backing up the general understanding of the field. In line with the constructivist strand of Grounded Theory (Charmaz, 2014), data collection and analysis proceeded in an iterative process. We conducted an inductive open coding to identify recurring topics, motifs and themes, which then informed further modification of the interview guide and the selection of possibly insightful conceptual lenses. The final steps consisted of an axial and later selective coding (Bryant, 2013).

### Four Domains of Boundary-Work around Ethics in DTC Neurotechnology Start-Ups

#### Deferring Hypotheticals: Shifting Specific Ethics Issues to the Future

When asked about potential ethical issues in the context of DTC neurotechnology, one leading scientist of a neurotech start-up responded that “if you start to talk about hypotheticals and you let it go too far into hypotheticals instead of focusing on the practical risks, you can get easily lost”. This quote illustrates a first distinction that actors in DTC neurotech start-ups regularly made when describing ethics implications of their products: between ‘actual’ challenges they are currently facing (such as data privacy and agency) and ‘hypothetical’ ethical issues (such as ′getting inside′ someone’s head or more fundamental philosophical debates) that their products might, or might not, raise in the future. Another interviewee argued that user safety is a central concern for them, but that questions of distributive justice are not yet, as the technology’s impact and large-scale dissemination are still limited: “I think it’s too early to really have serious ethical concerns about the devices that are out there […]. I think once the technology that Neuralink is building […], which has the potential to offer a greater brain capacity to some people who can afford it and not to other people on a really large scale or another order of magnitude of what we have now, then that would be a problem” (CEO & Founder, neurotech start-up).

This distinction of whether an issue qualifies as present or future is often made in conjunction with a differentiation between the ‘current’ technological capability and still unproven ‘future’ technological possibilities that may raise different ethics challenges, providing the basis for judging which respective ethical obligations to take seriously. The present-day ‘facts’ allow our interviewees to worry, for example, about safety or data privacy issues as real and legitimate concerns regarding DTC products currently available on the market. At the same time, it delineates issues that are not expected to actualize given the current impact of available technology, and for whom concern is neither warranted nor reasonable. For example, ostensibly metaphysical questions about personal identity or autonomy, privacy threats through far-reaching behavioral monitoring and surveillance, or a potential for discrimination through a slippery slope to human enhancement are often described as ‘hypothetical’ and hence deemed unfounded.

This line of reasoning further translates into imaginations about publics and consumers. Some interviewees seem to view public concerns about ‘hypothetical’ issues such as personal autonomy or mass surveillance as illegitimate or unreasonable. As previous research in STS has shown, in being attributed to technological illiteracy and a misunderstanding of the ‘actual’ technological functioning, the public’s concerns might be interpreted as irrational fear (Wynne, 1992). In that context, some interviewees used the implicit distinction between ‘actual’ vs. ‘hypothetical’ and ‘current’ vs. ‘future’ issues to differentiate products from dystopian visions and science fiction references, in which brain science has become a popular theme (Packer, 2014). By (dis-)connecting the ‘actual’ issues faced by neurotech start-ups from science-fiction television series like *Black Mirror*, possible wholesale concerns about DTC neurotechnology are rendered ‘hypothetical’ threats that only occur in fiction or with technological capacities expected to progress in the future. Thereby, ethical deliberation of particular problems is shifted to the future or the fictional, to the moment in which we “hit this kind of event horizon”, as one interviewee (CEO & Co-Founder, neurotech start-up) described it. Like in all acts of boundary-work, such demarcation is neither natural nor universal. Rather, it is a normative, situated proposition (in the sense that different start-ups will draw these demarcation lines differently depending on their particular position and politics in the field) and subject to collective negotiation with other actors in the field. Foregrounding a ‘current’ issue entails a value judgment as to what kind of ethical challenges are worthy of present-day consideration, and sets the agenda and scope of a company's responsibility.

Importantly, this does not mean that all ‘hypotheticals’ are automatically pushed into the domain of idle debates. Some actors described how they use them to think through possible future situations and acknowledge the role of science fiction as a basis to discuss desirable and undesirable socio-technical futures: “I think science fiction is probably the most forward-thinking type of engineering or creativity. […] I think it’s that sci-fi that has dystopian tones helps us stake out the future scenarios, that we don’t want to live, and design around that” (CEO & Co-Founder, neurotech start-up). Understanding and enacting ethics along such temporal dimension hence can serve strategic purposes for companies, which range from legitimizing attention (or inattention) to ethical problems in the present to actively shaping design processes toward ethically desirable future scenarios.

#### What's the Purpose? Drawing upon Ethics as Technology for the Public Benefit

A second strand of reasoning observed amongst interviewees unfolds along what could be considered traditional consequentialist lines. Many interviewees contend that DTC neurotechnology can be used for ‘good’ and for ‘bad’, and accordingly can bring positive or negative change to society (or lack any noteworthy effect altogether). In this line of reasoning, products are viewed as ethical and socially desirable depending on their purpose—or lack thereof: “[When] we look on the bright side of things, [Elon Musk] is helping people that maybe are paralyzed or can’t speak. So, there’s a good thing about it. But then there is also the line of doing crazy things which are not necessary or ethical” (CEO & Co-Founder, neurotech start-up). The quote illustrates how ethics within this dimension of boundary-work is imagined in terms that are closely tied to medicine: fulfilling medical and restorative needs, supporting the improvement of a health condition, mitigating suffering and enabling partaking in society is framed as ethical, forming a common anchor for positive applications. In contrast, interview participants expressed skepticism towards unethical DTC neurotechnology that lacks this purpose, need and justification. This includes, for instance, neuromarketing, “crazy” applications such as the scale of enhancement envisioned by Elon Musk, or “toys” that promise the remote control of a drone via the brain.

Emphasis on one’s commitment to idealist values helps to imagine ethical DTC neurotechnology as possible. As one CEO and Co-Founder of a neurotech start-up explained, “I would describe what we do, at least to a large extent, as social entrepreneurship, in the sense that our goal is to make technology accessible to people and that we really want to make sure that people have the opportunity to be happy with their brain. [We] believe in building an infrastructure where people can use neurotechnology in a high-quality and meaningful way, and not just buy some toys for home”. The potential benefits of DTC neurotechnology are often part of funding stories and mission statements, presenting powerful visions of how DTC neurotechnology will benefit society in the future and unlock untapped potentials and scales of human well-being. The potentials of neurotechnology to help individuals and society are positioned as a non-deniable legitimization for neurotechnological innovation, also on healthy brains and bodies and despite possible concerns that need to be overcome.

Framing ethics along the lines of neurotechnology's intended purpose and problem-solving capacity allows actors to actively link their innovation to those potential benevolent and visionary (future) applications and to establish their own businesses as virtuous. With the strong attachment to physical and psychological integrity pervading the actors’ understanding of desirable applications, medical ethics and the benevolence of healing professions surface as central motifs in this dimension of demarcation. Yet as noted before, DTC neurotechnology cannot officially claim to heal or cure and by definition is located outside the realm of medical technologies. However, as this domain of boundary-work shows, respective ideas of ethics play an important role. Jasanoff (2005a) noted that the positive connotations of medicine and the values it stands for (such as benevolence), have shaped not only bioethics but understandings of ethics beyond the medical domain as well. This halo of benevolence also seems to apply in the case of DTC neurotechnology, especially since many other neuroscientific and neurotechnological advances are squarely in the domain of medicine.

#### Safety First: Imitating Regulatory Review as Ethics

A third demarcation criterion through which our interview participants imagine and enact ethics is the criterion of safety. Safety is a well-institutionalized modality for evidencing ethics, as it constitutes one of the few central regulatory requirements for consumer electronics as well as a fundamental category in the regulatory process of medical devices. In the case of DTC neurotechnology, some of the safety risks that could potentially arise are skin irritation in contact with the devices or harms to psychological integrity. DTC neurotechnology products do not typically go through the process of medical approval, but—as stated above—do closely play on the boundary between medical and consumer devices as well as the safety standards that apply to other consumer electronics.

Our research finds that the interviewees’ reasoning on ethics often mirrors this regulatory vocabulary and logic. For example, when prompted about ethics, interviewees regularly referenced the technology’s invasiveness as a key criterion, i.e., the degree to which it intervenes in the body or the possible impact it can have on it. The following quote illustrates how safety, invasiveness and ethics connect in this domain of boundary-work: “[H]aving a USB-C port plugged into your brain and talking about how you can download your memory, or things like that, I’m like—whoa, ok. Where is the line we draw there? Of course, technology-wise it’s fascinating how far it can go. […]. But ethically, do we want to do that? […] So that’s why in the company, we always say that we’re doing things passively. We don’t do anything that’s invasive, because we don’t know what the long-term side-effects will be” (CEO & Co-Founder, neurotech start-up). The latent hierarchy in which passive (i.e., recording) devices are considered less problematic than active (i.e., stimulating) ones, and non-invasive stimulating DTC neurotechnologies less than invasive ones such as implants, closely mimics the medical regulatory process. Here, manufacturers have to provide evidence of safety according to a device’s risk, for which its invasiveness constitutes one determining factor in the regulatory context of our case study (FDA 2020; Radley-Gardner et al., 2016). The FDA, for instance, groups medical devices in three classes, stating that “classification is risk based, that is, the risk the device poses to the patient and/or the user is a major factor in the class it is assigned. Class I includes devices with the lowest risk and Class III includes those with the greatest risk” (FDA, 2020).^2^

Such framing reflects an understanding of ethics that is primarily reduced to safety as the mandatory legal requirement for market entry and accountability (see also Jasanoff, 1986). As one expert in the field of neurotechnology stated: “I presume that when the FDA says responsible, they mean safe”. Drawing a demarcation line according to the different degrees of invasiveness naturalizes the distinction between what is deemed ‘ethical’ or ‘unethical’ along the lines of bodily harm and makes it congruent with the regulatory distinction between ‘safe’ or ‘unsafe’. Companies may address ethics and put themselves on the ‘safe’ side by reference to the technological impact of their products according to medical criteria, which in the case of DTC neurotechnology are per definition low risk and, according to the FDA, applied for the purpose of general wellness (Kreitmair, 2019). On the one hand, this positioning helps start-ups to adhere to some relatively clear criteria in what is otherwise a rather unclear ethical landscape. On the other, this framing creates potential blinds spots for other ethical concerns that are particular to neurotechnology—such as EEG devices that are perfectly safe and non-invasive, but claim to provide an accurate snapshot of one’s mental state, which in turn might lead users to make changes in their behavior or self-understanding (Wexler & Thibault, 2019). Such concerns are part of the scenarios constructed amongst neuroethicists or in the media, but there currently exist no legal rules or sanctionable forms of self-regulation that ensure that they find practical, or proactive, consideration in business practice, as it is the case for safety.

#### A Business Based on Evidence: Ethics as Scientific Robustness

A last demarcation line for ethics identified in DTC neurotechnology start-ups pertains to questions of evidence and credibility, with boundary-work conducted around ‘sound science’, its norms, values and criteria. As one interviewee stated: “It is essential to not only do good science, but to show it” (President, neurotech start-up). For many actors, ethical behavior in this sense corresponds to avoiding overpromising and instead rather letting their products stand out based on their scientific soundness. Several interviewees approach this topic from a position of defensiveness in a Silicon Valley-centric sector that has been shaken up by some ‘bad apples’ in recent years. Beyond *Lumos Labs,* the prominent *Theranos* case serves as a key reference point for the field: The blood testing company and Silicon Valley posterchild had collapsed in 2018 after it emerged that their success was built on false claims and forged scientific evidence for the product’s performance. As warning flags that are proactively brought up by some interviewees, *Lumosity* and *Theranos* speak to an increased feeling of insecurity amongst actors suddenly in need of demonstrating ethical behavior and credibility, facing unprecedented public scrutiny towards the start-up scene and health technology in particular.

In this version of boundary-work, actors in the DTC neurotechnology industry are concerned about unsubstantiated scientific claims that lack a robust data base and methodology, fail to provide credible evidence for a product, or overpromise what the technology is capable of. Overstated claims might well be declared as such specifically because they lack scientific evidence, breaking with those norms considered to be at the core of the "ethos of science" (Merton, 1979), such as skepticism and disinterestedness. The issue here is not so much the violation of ‘science’ itself, but rather what it has come to stand for: objectivity, transparency and trustworthiness, and in turn the potential impression of dishonesty that might come from selling "snake oil" or "black magic" to vulnerable customers.

By understanding ethics in terms of robust, trustworthy science, the misleading of customers is portrayed as potential threat to the long-term reputation and image of the entire field of DTC neurotechnology. Beyond the ‘bad apples’ argument, entrepreneurs argue that overstated claims might feed into public fears of ‘too-powerful’ technology and lead to expectations in the market that cannot be met by the current state of technology. As one actor in the field explained: “For me, on the messaging and the marketing and the way we communicate, the way we educate people, we have an ethical responsibility of being honest of the limitations so that people don’t become overconfident. Because the problem with that is that it opens the door for snake oil vendors. People who are […] scamming if we’re too optimistic about the field” (Expert, neurotechnology field). In this understanding, irresponsible claims might generally undermine credibility and legitimacy which in turn causes mistrust amongst both investors who no longer want to invest in an emerging business and its promising visions, and regulators who no longer trust the industry’s capacity to regulate itself.

Through this framing, actors seek to establish credibility by relying on science as a social system that exhibits a set of recognizable and conventionalized rules and standards, such as practices of peer-review, providing scientific data and evidence, and being transparent about limitations. Start-ups furthermore recognize and assess other actors according to this logic. These findings are well in line with STS scholarship that has contributed to understanding how science has become a source of credibility and authority (Latour & Woolgar, 2008; Jasanoff 2005b). The findings in this fourth domain of boundary-work speak to the “wish, and ultimately the illusory belief that some standardized set of procedures called ‘science’ can provide us with an unimpeachable source of moral authority” (Postman, 1992, p. 162). Being regarded as scientifically robust—whether through the vocabulary used, the studies conducted by the company, or one’s ties to research institutions and studies in order to generate evidence—figures as a moral anchor and flagship, and contributes to an understanding of ethics as mutual oversight and self-regulation ‘through science’.

### Constructing Desirable Vanguard Visions through Ethics

The four dimensions of boundary-work discussed above (actual vs. hypothetical issues, good vs. bad purposes and consequences, consumer safety vs. medical risk or harm, and sound science vs. overpromising) illustrate how DTC neurotechnology start-ups conceptualize and enact ethics in their specific settings and to advance their particular interests. Our analysis shows the entrepreneurs’ considerations of optimistic, revolutionary visions of their technologies on the one hand, and their careful, humble acknowledgement of certain ethical hazards and risks on the other. It is between such “technological hubris” and “humility” (Jasanoff, 2003) that we locate our analysis of how ethics in the emerging sector of DTC neurotechnology innovation comes to be framed, mobilized and enacted.

The dynamics we observe correspond to what Hilgartner (2015) identifies as a struggle over competing *vanguard visions* that speak to different interpretations of the public good in relation to an emerging technology. In Hilgartner’s work, the concept of *sociotechnical vanguards* denotes “relatively small collectives that formulate and act intentionally to realize particular sociotechnical visions of the future that have yet to be accepted by wider collectives, such as the nation” (p. 34). Vanguards engage in distinct competitive strategies that make their visions not only imaginable by but also plausible and reasonable to wider publics.

Based on our previous analysis and in considering start-ups in the DTC neurotechnology sector as sociotechnical vanguards, we suggest that ‘ethics’ figures as an element that contributes to mediating how the actors in our study articulate visions of successful technologies and desirable futures—and make them acceptable, plausible and reasonable. Instead of being only perceived as a resource-consuming potential threat to business sustainability, employing ethics also provides a competitive advantage for emerging neurotech companies. The way in which actors mobilize ethics to make certain visions plausible can be conceptually grasped through Hilgartner's (2017) work on *knowledge-control regimes*, described as more or less formalized “structures that allocate entitlements and burdens pertaining to knowledge” and that rely “on many kinds of legal and quasi-legal mechanisms as well as on collectively understood templates for engaging in ‘guided doings’” (p. 9). Through the strategies of boundary-work, actors themselves create such a mapping of entitlements and burdens concerning the ethical handling of DTC neurotechnology. They define potential parameters of control—the scope of ethical issues, what to do about them, and their own responsibilities towards them—in a way that we describe as a form of displacement through which they establish desirable technology trajectories as a relationship between the present and the future.

Felt et al. (2009) used the term of “displacement strategies'' (p. 360) in the context of a public participation exercise to describe the shift of ethical reasoning away from the here and now. We encountered a similar displacement strategy in the form of a deferral of certain ethical ‘hypothetical’ issues and problems to the future. But the future is also mobilized in the name of the technology’s potential: promissory assertions about neuro-futures such as healthier and happier lives for everyone, accompanied by a democratization of neurotechnology drive this vanguard vision forward and create its legitimacy in the present (Borup et al., 2006; Brown & Michael, 2003). At the same time, futures governed by the wrong kind of neurotechnology, or no neurotechnology at all, are evoked as undesirable scenarios to which actors can provide an effective alternative. This enactment of ethics represents a key device for linking a selective interpretation of the present to an (equally selective) interpretation of the future rendered desirable.

In turn, another form of displacement can be perceived regarding the framing of ethics relevant to the here and now: the delegation of ethical considerations and control to already existing, validated forms of expert knowledge and their respective institutions. Our interviewees not only stake out a manageable scope of ethical activity defined by the societal and legal expectations towards safety and fair marketing, but also the respective sets of rules and authority, such as the law in the case of safety (e.g., Jasanoff, 2005c) and science in the case of evidence-base (e.g., Latour & Woolgar, 2008). The fallback onto existing forms of expertise has also been observed beyond the field of DTC neurotechnology and is key to the enactment of a vanguard vision, which is “more likely to gain traction if it is tied to entities and expectations familiar enough to provide an intelligible guide to the imagined future” (Hilgartner, 2015, p. 40). It is not surprising that legal requirements play a central role in ethical deliberations and practical imperatives, and it reminds us that while start-ups “seek to make futures, …they cannot make them simply as they please [..] but do so using vocabularies and practices already given from the past” (ibid, p. 50).

The strategies of boundary-work and our suggestion of connected ethical displacements carve out a certain scope of governance obligations for start-up actors, paving the way for socio-technical transformations and respective vanguard visions premised on the assertion that DTC neurotechnology innovation will be beneficial for and can indeed be trusted by individuals and societies despite currently loose regulation. We argue that through the framing of ethics analyzed here, start-ups establish DTC neurotechnology as ethical in itself and propose their own view of how DTC neurotechnology and emerging ethical issues should be handled. Through the anchoring of ethics in safety and evidence-base, actors take control of the associated present challenges, fulfilling their role as responsible and considerate innovators within this designated scope. This, in turn, allows for the deferral of ethical issues that are ‘not yet critical’, and thought further, might set the argumentative basis for avoiding stricter regulation in the present and the unhindered pursuit of innovation. At the same time, we should keep in mind that many of our interviewees verbalized the desire for clearer guidelines in the field that would give them more certainty in addressing current ethical, legal and social issues, as well as public scrutiny.

## Conclusion

The ethics of neurotech innovation generally, and of DTC neurotechnology devices in particular, are currently problematized from various perspectives, including scholarship and policy-making on *neuroethics, RRI,* and *CSR*. Our empirical case study exemplifies how ethics frameworks introduced by expert communities do not necessarily reflect the 'ethical realities' described by the actors who are involved in private-sector R&D and commercialization and who pursue their own strategies of defining ethics according to their own instrumental needs, envisioned purposes and contexts. Based on our findings, we suggest that the construction of ‘ethics’ could play a key role in establishing credibility, legitimacy, and autonomy in a still unsettled and highly contested field, and thereby contributes to making the future-looking vanguard visions around DTC neurotechnology start-ups plausible. We characterize the framing of ethics through the actors’ strategic boundary-work and connected demarcations as two forms of displacement—the ‘deferral’ of ethical challenges and benefits to the future, and the ‘delegation’ of ethical reasoning to established knowledge regimes of ethical oversight—which allows them to construct plausible and desirable technology trajectories from the present into the future. From this perspective, ethics becomes a key ingredient of nascent knowledge-control regimes (Hilgartner, 2017) where the power to shape a specific understanding of ethics at once allocates rights and responsibilities and legitimizes certain visions of desirable socio-technical futures and neuro-innovation practices.

In order to better account for the politics of making technology *and* making its ethics, and to move from a prescriptive to an empirically grounded approach, our findings align with a recent paper by Pfotenhauer et al. (2021), suggesting that private sector governance in neurotechnology could greatly benefit from a co-development of regulations, including “alliances of companies, policymakers, academics and citizens” (p. 3). Here, co-creative and deliberative approaches should attend to the manifold ethical implications not only as a speculative ethics of the future but of present ethical practices and visions advanced by start-ups and the technology industry.

## Footnotes

1 Both in the US and the EU, which is the regulatory context we considered in our research.

2 Class I: Devices present minimal potential for harm to the user / Class 2: Higher risk than class 1; Class 3: Devices usually sustain or support life, are implanted, or present potential unreasonable risk of illness or injury (FDA, 2018).
